# Is Stem Cell Therapy an Answer to Heart Failure: A Literature Search

**DOI:** 10.7759/cureus.5959

**Published:** 2019-10-22

**Authors:** Javaria Tehzeeb, Anam Manzoor, Munis M Ahmed

**Affiliations:** 1 Internal Medicine, Mayo Hospital, King Edward Medical University, Lahore, PAK; 2 Internal Medicine, St Mary Mercy Livonia Hospital, Livonia, USA

**Keywords:** regenerative medicine, heart failure, stem cell transplantation, cell therapy, transplantation, heart failure/mortality, stem cells and ischaemic heart disease

## Abstract

The heart is one of the most industrious organs in the human body. It starts beating in the first few weeks of embryonic life and keeps pumping blood till death. This organ can host a range of diseases as well. Some can hamper the vasculature, while others can affect its electrical activity, the heart valves, etc. All these conditions can lead to end-stage failure where it can no longer meet the requirements of the body’s milieu. This imbalance between supply and demand leads to an array of symptoms. Medical management can reduce these clinical effects and possibly prolong the life expectancy in such patients. However, prescription medications can also have their own adverse effects. This necessitates that each line of treatment should be assessed on a risk vs benefit basis. The conventional approach has been to try and slow down the progression of heart failure (HF). However, the inception of stem cells in the management of HF has the potential for reversal of this pathology. Keeping this in view, many studies and trials are under process. To turn the clock back on the HF, before complications set in or get out of control, is the main focus of the time. This article attempts to evaluate various studies about stem cell therapy (SCT) and highlight the important aspects of this novel modality in changing patients' lives.

## Introduction and background

The heart is the first functional organ to develop in the human body, and it continues to perform its work as long as a person lives. This organ can also host various ailments. Some of these conditions originate from a compromise in its vascular supply, while others may affect the heart valves or its conduction system. Moreover, cardiac conditions can also develop due to certain systemic diseases such as connective tissue disorders. Any disease that decreases the filling or pumping action of the heart leads to an array of symptoms, collectively termed as heart failure (HF). There is a lack of consensus on a single definition for HF [1]. In simple terms, HF implies that the heart is not pumping well enough for normal body functioning, which may have a negative impact on the other organs.

HF is an epidemiological concern worldwide. In the United States alone, 6.2 million people were reported to suffer from HF between 2013 and 2016 [2]. One in three patients with HF is expected to survive for five years [3]. Various treatment modalities are available, among which medical management has been the primary approach for a long time. The drugs traditionally used include diuretics, angiotensin-converting enzyme inhibitors, beta-blockers, digoxin, etc. [4]. With the increase in the severity of HF, more aggressive treatment options have to be employed. Hence, patients with higher stage disease become candidates for more advanced treatments such as mechanical assist devices and transplantation. Heart transplant has been considered the gold standard treatment for all causes of HF, with a 10-year survival rate of more than 50% documented in the recipients [5-6]. However, there are limitations to this treatment such as a disproportion between demand and the availability of donors. Recently, stem cell therapy (SCT) has been considered as a promising alternative to conventional treatments. The use of stem cells for the replacement of damaged heart tissues can help improve functioning [7]. In addition, these cells have shown a beneficial role in stimulating neovascularization and preventing myocardial cell death. They can also limit the need for medical management as well as surgical procedures in patients suffering from HF and improve their quality of life substantially.

## Review

The "stem cell" is the term used for a cell lineage having the potential to differentiate into multiple other types of cells. Mesenchymal stem cells (MSC) are the most important members of this lineage from a clinical perspective. Many tissues in the body host these multipotent cells, the largest source being the bone marrow. Some specific locations in the body are expected to harbor this population as well, such as adipose tissue, lungs, and blood vessels [8]. Among these, the vascular endothelial lining is of interest from a cardiovascular point of view. Based on their property of rapidly dividing and maturing into more differentiated forms, stem cells are considered as a useful tool in the management of HF. Extensive studies are being conducted to prove their usefulness and practical application. Since the last few decades, they have been transplanted into diseased cardiac tissues and are expected to replace the damaged and scarred areas with normal myocardium [7]. We now know that their benefits range beyond their innate differentiating abilities. The newer studies point out that the most significant aspect of stem cells is their capability to secrete various hormone-like substances and growth factors. These substances chemically act on areas such as the damaged myocardium and endothelium of vessel wall and yield advantageous responses. These paracrine effects surpass their restorative function in magnitude and benefits. For example, they can prevent cell death and cause new vessels to sprout to supply the ischemic areas [9-10].

Mononuclear cells (MNC) are an even larger population of stem cells. Both bone marrow-derived MNCs and MSCs have been independently applied in the management of HF. Their chemical behavior has been studied to be different from each other. MSCs are more likely to have a regenerative and vascular proliferative capacity, while MNCs act more by triggering favorable forms of inflammation [11]. This observation adds a perspective of paramount significance to SCT, suggesting that this therapy can yield different responses depending on the type of cells used. Results also vary depending on whether cells come from the patients themselves or a donor (autologous vs allogeneic sources). All of these add up to imply that SCT is a complex and multi-dimensional line of treatment and multiple factors play a role in determining its benefits and risks.

A limited randomized control trial (RCT) published in 2005 studied 20 patients with ischemic HF and left ventricular ejection fraction (LVEF) documented as less than 35% of normal [12]. Stem cells were delivered to the subepicardial areas of the patients in the experimental group. It showed an increase in LVEF from a pretreatment value of 29.4% of normal to 46.1% in the patients with ischemic HF receiving autologous SCT plus coronary artery bypass grafting (CABG), as opposed to an increase from 30.7% to 37.2% in the control group that received only CABG. No adverse effects were observed in either group. Clinically, this was a notable finding in favor of SCT.

A 2006 study recruited patients who had a history of acute myocardial infarction (MI) successfully treated with thrombolysis or anticoagulation [13]. The use of stem cells in these patients manifested a significant reduction in cardiovascular mortality and recurrence of acute ischemic events with a concurrent increase in LVEF. The patients with LVEF lower than the median value showed the greatest improvement in function. However, in this study, stem cells were introduced into coronary arteries.

The reason for mentioning the intracoronary or subepicardial routes is that these are the safer techniques of stem cell delivery. The other methods that involve direct injections into the myocardium pose a risk of local injection-site damage and arrhythmias [14]. These arrhythmias may be triggered by the inflammation and scarring from injections. Moreover, the integration of grafted cells may have some physical and electrophysiological effects that increase the arrhythmogenicity. These risks can be mitigated by choosing safer routes to deliver stem cells. Another point worth mentioning is that the risk of arrhythmias is most strongly associated with stem cells of skeletal muscle origin. "The Myoblast Autologous Grafting in Ischemic Cardiomyopathy" (MAGIC) trial was a clinical study that was able to study this association. It found no improvements in LVEF with the use of stem cells of skeletal muscle origin [15]. This trial also found arrhythmias in the stem cell treatment group as opposed to controls. This implies that skeletal muscles are not an efficacious source of stem cells for use in HF.

Another clinical trial in 2010 "The Acute and Long-term Effects of Intracoronary Stem Cell Transplantation in 191 patients with Chronic Heart Failure: the STAR-heart Study" involved intracoronary injections of stem cells in patients with ischemic HF and low LVEF [16]. The cells were obtained from the bone marrow of the same patients to whom they were delivered (autologous). Data analysis at up to five years after the initiation of this treatment showed that SCT led to an increase in the New York Heart Association (NYHA) functional class and LVEF in the treated patients. In addition, a decrease in the left ventricular preload, end-systolic volume (ESV), systolic wall stress, area of infarct, and long-term mortality was noted. Only routine procedural side effects were observed. Arrhythmias were found to be lower in the stem cell group. Based on these findings, this study found SCT to be safe and beneficial in HF patients with a history of old MI who had initially undergone some form of revascularization or anticoagulation. This study especially points out the improved mortality profile seen with SCT, attributing it to both improved cardiac performance and reduced occurrence of arrhythmias.

A study titled "Comparison of Allogeneic vs Autologous Bone Marrow-derived Mesenchymal Stem Cells Delivered by Transendocardial Injection in Patients with Ischemic Cardiomyopathy: the POSEIDON Randomized Trial" compared autologous and allogeneic MSCs in ischemic HF patients to identify the type of donor stem cells to be preferred [17]. Both types of MSCs demonstrated beneficial effects in the form of reduction in the area of infarct after acute MI. The parameters of quality of life showed an improvement, more so in the autologous MSC group. One of the most important targets of HF management is to prevent harmful cardiac remodeling. This study was able to show that SCT is beneficial in this respect and also showed a reduction in disadvantageous cardiac structural changes. However, no improvement was seen in markers of left ventricular function like LVEF. Antibody response to allogeneic MSCs was small in magnitude and there was no acute immune rejection. Significant adverse events, especially arrhythmias, were found lower in the allogeneic MSC group. These observations imply that both types of MSCs are safe to use in patients with HF. Moreover, it explains that the risk of immune rejection or damage with the use of allogeneic MSCs is too low to jeopardize their clinical usefulness.

One of the recent ways in which stem cells have been used is called cardiopoiesis. It consists of subjecting stem cells to procedures that predetermine their fate to some extent as to which type of cells they will mature into [18]. The "Cardiopoietic Stem Cell Therapy in Heart Failure" (C-CURE) trial is one of the first trials of its kind started in 2013 based on this principle [19]. Analysis at two years showed an improvement in the cardiovascular functioning status using such cells in ischemic HF. The objective findings included that this treatment increased LVEF from 27.5% of normal to 34.5 %. Also, a lower left ventricular ESV was noted in the stem cell group, which decreased by 24.8 ml, while only an 8.8-ml reduction was seen in controls. Any variation in harmful cardiac events between the two groups was found to lack statistical significance. Other aspects of cardiac function and quality of life were assessed by employing measurements such as the NYHA functional class and hospital admissions. All of these parameters showed an improving trend with SCT.

The beneficial aspects highlighted in the C-CURE trial prompted larger studies on this type of SCT. One of the largest trials based on this concept is the “Congestive Heart Failure Cardiopoietic Regenerative Therapy” (CHART-1) trial in 2015 [20]. The latest analysis of its findings was done at 39 weeks since the start of this trial by following up 120 patients treated with these cells and 151 patients who were given a sham procedure. The results show that this trial shares some of the findings of the C-CURE trial. For example, no excess harmful effects were reported in the stem cell group. It especially points out the lower incidence of sudden cardiac deaths in this group of patients. Throughout the 39 weeks, an improving trend was observed in the cardiac function of patients with left ventricular end-diastolic volume (LVEDV) of 200-370 ml who received SCT. Overall, these trials affirm that SCT is safe and has long-lasting benefits. The most significant aspects of these studies are their use of modern technology and long follow-up, thereby making them practical. The quality of evidence also increases when consistent benefits are seen over a long duration. Moreover, they can help indicate rare but significant adverse events, which is very important to assess before SCT becomes a widely used modality.

The trials discussed so far appear to stress that stem cells are proving to be a reliable therapy as they are safe and yield better results as compared to past medical and surgical options. However, other studies indicate that the response to SCT can vary from patient to patient, and the results are neutral or inconsistent [21-22]. In the majority of trials, autologous stem cells were used. However, many patients with HF lacked usable populations of stem cells [23]. These findings raise questions about difficulties in predicting prognosis after the use of SCT. They also stress the importance of sources of stem cells, comparing them to find which is better and studying the methods of their retrieval.

A “Phase II Dose-Escalation Study of Allogeneic Mesenchymal Precursor Cells in Patients with Ischemic or Nonischemic Heart Failure” conducted in August 2015 has studied the dose-response aspects of MSCs, forming three groups of 20 patients each. Fifteen patients in each group received one of the three selected doses (25, 75, or 150 million cells) of MSCs [24]. The remaining five patients underwent a mock procedure. The MSCs used in this trial were processed by immunologic selection methods. The purpose of this selection procedure was to study if immune responses affect SCT and to establish the immunological safety profile of MSCs. The cells were delivered to patients by transendocardial injections. The results from this study highlight the beneficial effects of SCT in preventing major adverse cardiac events including death from a cardiac cause, acute coronary syndrome, or need for revascularization. Cardiac adverse events were observed in 22% of patients who received MSCs and 33% of controls. HF-associated cardiac events were found to be lower in all three dose cohorts.

Antibodies with donor antigen specificity were found in 11% of stem cell-treated patients but caused no noticeable adverse effect. Small sample size and predominance of ischemic heart disease in patient groups were limitations of this study. Earlier studies had focused on regenerative aspects of SCT [25-26]. However, this study puts more emphasis on their paracrine effects. This solidifies the modern approach to SCT that these cells are not only useful because they replace the dead or damaged tissue as they also improve the function of surviving tissue, reduce harmful remodeling, and stimulate secretion of cytokines and growth factors. All of these effects contribute to the clinical improvement seen in treated patients.

Endothelium forms a single-layered, squamous epithelial lining of vessel walls. It forms a mechanical and charge barrier between intravascular and interstitial compartments. It also produces various cytokines and growth factors that affect vessel caliber, coagulation, etc. Endothelial dysfunction forms the pathophysiologic basis of many diseases. These include atherosclerosis, diabetes mellitus, and complications [27]. Some physical features of HF are also attributed to disturbed functions of endothelium, leading to an increase in the resistance of peripheral vessels, i.e., an increase in afterload for a failing heart [28]. The endothelium also harbors some lineage-specified progenitor cells that can serve regenerative purposes. MSCs have shown to be useful in this aspect as well. They have been found to counter the endothelial dysfunction by inhibiting harmful inflammatory processes. They inhibit scar formation and enhance the sprouting of new vessels in diseases and damaged areas [29]. It has been suggested that endothelial progenitor cells (EPC) can be stimulated to proliferate by MSCs and help counter the endothelial dysfunction [30].

A clinical trial titled “Allogeneic Mesenchymal Stem Cells Restore Endothelial Function in Heart Failure by Stimulating Endothelial Progenitor Cells” studied this relationship [31]. It showed that endothelial progenitor cells were markedly reduced in patients with HF as opposed to healthy controls. The EPC counts were seen as 4 (± 3) colony-forming units (CFU) in HF patients but 25 (± 16) CFUs in healthy controls yielding a p-value of less than 0.0001. In this study, it was observed that allogeneic MSCs enhanced this EPC count at three months follow-up. SCT was also associated with lower vascular endothelial growth factor (VEGF) levels, compared to autologous stem cells (*P* = 0.0012). Based on these findings, this study confidently asserts the advantageous effects of allogeneic MSCs on endothelial cells despite the sample size and matching limitations.

One of the inevitable limitations of SCT is that MSCs are expected to lose some efficacy due to aging [32]. Studies suggest overcoming this limitation by obtaining stem cells allogenically from healthy donors instead of autologous cells from HF patients who may be old and have additional co-morbidities.

In the "Bone Marrow-derived Mesenchymal Stromal Cell Treatment in Patients with Severe Ischemic Heart Failure: A Randomized Placebo-controlled Trial (MSC-HF trial)" conducted in Europe in 2015, autologous bone marrow-based stem cells were used in ischemic HF patients [33]. Approximately 55% of patients reached the primary efficacy checkpoint at six months. Left ventricular ESV was found to be 7.6 mL lower with a *p*-value of 0.001 in the stem cell group as opposed to controls. SCT also showed an increase of 6.2% in LVEF (*p *< 0.0001), 18.4 mL in stroke volume (*p* < 0.0001), and 5.7g in the mass of myocardium (*p* = 0.001). These parameters have proven to strongly reflect cardiac function status, and any improvement in these values suggests long term survival benefits in HF patients [34-35]. MSC-HF trial used some of the same surrogate measurements to assess efficacy secondarily as were used in the C-CURE trial (like NYHA functional class) and found them to be comparable between the treatment group and the controls. The adverse effects of SCT were found to be negligible in this trial. Also, fewer patients in the SCT group needed admission to the hospital due to the worsening of angina symptoms.

Treatment-resistant angina has been seen to have a similar improvement in a previous study in 2013 [36]. Outcomes showed significant effects of the dosage of stem cells, with maximal improvements associated with maximal dosage used. Although previously considered safe, patented catheter injections showed two significant adverse events in this trial [36-37].

A phase 3 RCT titled " The Effect of Intracoronary Infusion of Bone Marrow-derived Mononuclear Cells on All-cause Mortality in Acute Myocardial Infarction: Rationale and Design of the BAMI Trial" has been initiated at an international level. The results of this RCT indicate that the patients with low LVEF (less than or equal to 45%), who had previously undergone reperfusion successfully after acute MI, showed a decrease in mortality after receiving autologous bone marrow MNCs in comparison to the patients that received standard medical treatment only [38]. It can be seen that this is one of the similar results in a series of clinical trials that show statistically significant improvement in LVEF and reduced mortality with SCT and this evidence seems to be getting stronger in the more recent trials.

In another RCT, the patients who were on left ventricular assist devices were weaned temporarily and given SCT to determine how these patients responded to this therapy. No evidence of benefit was observed in these patients at one-year follow-up [39]. This indicates two things:

1) SCT may not be able to deliver benefits in situations where a more acute management option is needed.

2) SCT is not an absolute replacement for mechanical or surgical procedures in patients already on the latter treatments.

A German 2012 review article suggested that phase 1 and 2 clinical trials on stem cell-based management of HF have been able to establish their safety and efficacy. Yet, more multicenter phase 3 trials are needed before any further conclusions are drawn regarding which stem cell type is most useful and at which stage of HF should they be introduced to be most effective [40].

Most of the clinical trials we have discussed seem to focus on HF due to ischemic causes. However, non-ischemic HF is not exempt from the benefits of SCT. A review article published as recently as 2015 gathered evidence to suggest that the use of the cluster of differentiation (CD) 34+ stem cells in non-ischemic causes of HF also improves cardiac functioning and symptoms [41]. This review goes on to reiterate that in cases of ischemic HF, cells obtained from the cardiosphere reduce the amount of scar enhance the amount of functioning tissue and increased wall thickness in the myocardium. It explains that MSCs are useful in HF irrespective of their allogeneic or autologous sources. However, the article also points out that it may not be feasible to lay down the same stem cell regime for patients with different causes of HF because the variability in the spectrum of the disease has been associated with inconsistent observations. Hence, this article urges a need for personalized treatment regimes.

## Conclusions

HF can lead to a compromise of various bodily functions leading to catastrophic outcomes. Various techniques have been instituted to overcome the harmful effects and improve functioning in patients suffering from this problem. This review has gathered that many studies favor the benefits of SCT showing that it not only improves the primary cardiac condition but also helps to avoid further decline in the functionality of the heart. The advent of this promising modality can benefit many individuals who either are not candidates for surgery or do not benefit from medications. However, inconsistent results and inter-person variability in outcomes of the existing studies on SCT are still areas of concern and warrant an extension of the spectrum of clinical studies in this field.
